# The Value of TyG-Related Indices in Evaluating MASLD and Significant Liver Fibrosis in MASLD

**DOI:** 10.1155/cjgh/5871321

**Published:** 2025-03-13

**Authors:** Haoxuan Zou, Jiejie Xie, Xiaopu Ma, Yan Xie

**Affiliations:** Department of Gastroenterology and Hepatology, West China Hospital, Sichuan University, Chengdu, Sichuan, China

**Keywords:** AUC, DCA, IDI, MASLD, NHANES, NRI, smooth curve fitting, TyG, TyG-BMI

## Abstract

**Background:** Triglyceride glucose (TyG) and its related index (TyG-body mass index, TyG-BMI) are recognized as markers for nonalcoholic fatty liver disease (NAFLD), but their associations with metabolic dysfunction-associated steatotic liver disease (MASLD) and significant liver fibrosis (SLF) risk are less studied. Therefore, this study explores the effectiveness of these indices in assessing MASLD and SLF risk in the U.S. population.

**Methods:** Utilizing data from the National Health and Nutrition Examination Survey (NHANES), a cross-sectional study involving 5520 participants from the general population was performed. This research measured demographic, anthropometric, biochemical, comorbid, and lifestyle characteristics, all of which are considered risk factors for MASLD/SLF.

**Results:** Upon controlling for confounding variables, only the TyG-BMI was found to have a consistent positive association with the risk of MASLD and SLF. Specifically, for each standard deviation increase, the odds ratio (OR) and 95% confidence interval (CI) were 4.44 (3.64–9.26, *p* for trend < 0.001) for MASLD and 2.48 (2.15–2.87, *p* for trend < 0.001) for SLF. Significant interactions were identified among age, sex, and the risk of MASLD associated with the TyG-BMI. The TyG-BMI also had a significant threshold effect on the risk of MASLD at a cutoff point of 180.71. Furthermore, the area under the receiver operating characteristic curve (AUC) revealed that the TyG-BMI better predicted the risk of MASLD and SLF (AUC 0.820, 95% CI 0.810–0.831; AUC 0.729, 95% CI 0.703–0.756, respectively). In addition, the integrated discrimination improvement (IDI), decision curve analysis (DCA), and net reclassification index (NRI) also demonstrated the satisfactory predictive ability of the TyG-BMI.

**Conclusions:** Within this large dataset, the TyG-BMI was independently associated with both the MASLD score and the SLF in the MASLD cohort. Its predictive efficacy consistently surpassed that of TyG and other noninvasive models, indicating that TyG-BMI has potential for the early identification of MASLD and SLF risk.

## 1. Introduction

The estimated prevalence of nonalcoholic fatty liver disease (NAFLD) exceeds one-third of the global population, which imposes a substantial health burden and economic strain on all nations [1, 2]. In 2020, experts from 22 countries suggested renaming NAFLD to metabolic dysfunction-associated fatty liver disease (MAFLD) on the basis of clear diagnostic criteria independent of other liver diseases [3]. However, the term “fatty” in MAFLD remains stigmatizing, and the classification regarding fatty liver and alcohol use is unclear, raising concerns about potential confusion in identifying causes [4]. As a result, the recent Delphi Consensus Statement, led by several national hepatology societies, has again recommended that NAFLD be renamed MASLD, which is a positive and nonmisleading description of the disease, and this latest renaming is a much more comprehensive and nuanced delineation of the disease [5]. As steatotic liver disease (SLD) advances to various stages of liver fibrosis, it may progress to cirrhosis or hepatocellular carcinoma. Liver fibrosis, particularly SLF, has been extensively documented as being strongly correlated with poor prognosis and is regarded as a good prognostic indicator for SLD [6–8]. Globally, chronic liver disease associated with hepatic fibrosis is also associated with increased mortality and morbidity [9]. Thus, early identification of patients at high risk of MASLD and significant liver fibrosis (SLF) in MASLD will help us to intervene in a timely manner to control their progression, greatly improve their prognosis, and reduce the medical burden.

Insulin resistance (IR), which is characterized by decreased insulin sensitivity and impaired glucose processing, plays a key role in NAFLD development [10, 11]. The triglyceride glucose (TyG) index, which is based on triglyceride (TG) and fasting plasma glucose (FPG) levels, is a reliable alternative measure of IR [12, 13]. In addition, some studies have shown that the TyG-body mass index (TyG-BMI), consisting of the TyG and BMI, is superior to the TyG itself in predicting IR given the close link between IR and obesity [14, 15]. The limited studies examining the link between these indices and NAFLD/MAFLD have shown varying diagnostic efficacies, mostly focused on comparing the area under the receiver operating characteristic curve (AUC) [15–22]. Furthermore, evidence suggests that IR is linked to hepatic fibrosis and has predictive value for its occurrence [23–26]. IR facilitates the transport of free fatty acids (FFAs) to the liver via multiple pathways. When the levels of FFAs surpass the oxidative capacity of cellular mitochondria, lipotoxicity ensues, impairing insulin signaling, inducing oxidative damage, and promoting inflammation and the progression of fibrosis [27, 28]. The onset of IR is recognized as a significant contributor to hepatic fibrosis and is also a critical factor in the development of liver fibrosis [27, 29]. Very few articles have explored the link between TyG-related indices and hepatic fibrosis in NAFLD patients [16, 30]. To our knowledge, no prior studies have examined the link between these indices and SLF in MASLD, nor have they explored whether TyG-related indices can identify SLF risk in MASLD.

This study aimed to investigate the connection between TyG-related indices and MASLD/SLF in U.S. adults, assess their predictive value for MASLD and SLF risk, and offer new prevention and treatment strategies for MASLD.

## 2. Materials and Methods

### 2.1. Data Source

The study used data from the NHANES 2017–2020.3, with all participants signing consent forms. The survey protocol received approval from the National Center for Health Statistics Research Ethics Review Board (NCHS IRB/ERB: 2011-17 and 2018-01). As part of the study, we followed the STROBE guidelines for reporting this population-based observational study [31].

### 2.2. Data Collection and Definitions

The NHANES served as the main data source, with details on its variables available in the online Supporting Information. The Supporting Information includes definitions of lifestyle factors, demographics, and comorbidities such as smoking, racial status, alcohol use, diabetes, and hypertension. It also provides formulas for calculating indices such as TyG [13], TyG-BMI [14], the visceral adiposity index (VAI) [32], the Zhejiang University index (ZJU) [33], the hepatic steatosis index (HSI) [34], the fibrosis 4 index (FIB-4) [35], the noninvasive Koeln–Essen index (NIKEI) [36], and the NAFLD fibrosis score (NFS) [37].

In this study, a controlled attenuation parameter (CAP) threshold of ≥ 274 dB/m was used to denote the presence of significant hepatic steatosis, whereas an liver stiffness measurements (LSM) threshold of ≥ 8.2 kPa was employed to indicate SLF, as supported by previous research [38]. MASLD was defined as the presence of significant liver steatosis and at least one cardiometabolic risk factor (CMRF), excluding individuals with high alcohol intake (over 140 g weekly for females and over 210 g for males) or other causes of hepatic steatosis [5].

### 2.3. Statistical Analysis

Statistical analysis was conducted with R 4.3.2 and Empower 4.2, using a 2-sided *p* value of < 0.05 for significance. Following the NHANES guidelines, we used 2-year sample weights from the NCHS analysis guide to improve our study's representativeness [39]. Continuous variables are shown as mean (SE), with *p* values from the weighted linear regression model. Categorical variables are given as % (SE), with *p* values from the weighted Chi-square test. Sample weights were included in all estimates.

The study developed three logistic regression models, adjusting for covariates significantly linked to MASLD or SLF (*p* < 0.1) or those altering the TyG-related indices' effect size on MASLD or SLF by more than 10%. Multicollinearity in the logistic regression model was assessed using the variance inflation factor (VIF), with all variables showing a VIF below 10, indicating no significant multicollinearity. For MASLD, the crude model had no adjustments for confounding factors; the minimally adjusted model accounted for sex, race, age, alcohol consumption, LSM, hypertension, and diabetes; the fully adjusted model further included adjustments for alanine aminotransferase (ALT), aspartate aminotransferase (AST), alkaline phosphatase (ALP), albumin (ALB), γ-glutamyl transpeptidase (GGT), high-density lipoprotein cholesterol (HDL), and estimated glomerular filtration rate (eGFR). For SLF in MASLD, the crude model had no adjustments; the minimally adjusted model accounted for age, sex, race, alcohol use, diabetes, and hypertension, and the fully adjusted model included further adjustments for ALT, AST, GGT, ALP, HDL, and CAP.

Subgroup analysis of TyG indices was performed based on the basis of age, race, sex, hypertension, and diabetes. Smooth curve fitting was used to detect nonlinear relationships. If it is found, a two-piecewise linear regression model identifies the threshold effect on MASLD/SLF, clarified by a smoothing plot. A log-likelihood ratio test (LRT) then compares the one-line and two-piecewise linear regression models [40]. Furthermore, we assessed the predictive power of TyG-related indices via ROC analysis based on “pROC” package and compared them with existing models via the Delong method [41]. We also evaluated the models' clinical utility through integrated discrimination improvement (IDI), decision curve analysis (DCA), and net reclassification index (NRI) based on “rmda” and “PredictABEL” packages [42–44].

## 3. Results

### 3.1. Baseline Characteristics of the Participants

**Figure 1:**
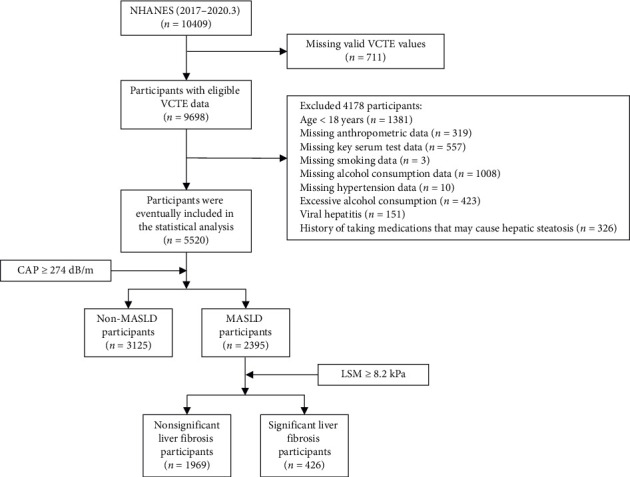
Diagram of the study design flow.

As shown in Figure 1, after excluding participants missing key variables, 5520 subjects from the 2017–2020.3 NHANES cycle were included. Among them, 2395 (weighted proportion, 42.43%) were diagnosed with MASLD, and 426 (weighted, 17.53%) had SLF within MASLD. The participants in this study included 2733 women (weighted, 50.66%) and 2787 men (weighted, 49.34%), with a weighted mean (SE) age of 46.79 (0.67) years.

Table 1 presents the weighted demographic, anthropometric, biochemical, comorbidity, and lifestyle characteristics categorized according to MASLD status. The weighted proportion of males and Hispanic individuals in the MASLD group was greater than that in the non-MASLD group. Additionally, subjects with MASLD were significantly older and had a greater waist circumference (WC), BMI, CAP, LSM, FPG, TG, total lipoprotein cholesterol (TC), AST, ALP, ALT, globulin (GLB), GGT, uric acid (UA), creatinine (CRE), and percentages of participants with diabetes and hypertension. Moreover, subjects with MASLD demonstrated significantly lower levels of ALB, eGFR, and HDL. Furthermore, individuals with MASLD presented significantly elevated TyG and TyG-BMI values. Supporting Table 1 shows the weighted baseline characteristics according to SLF status in MASLD.

### 3.2. The Relationship Between the TyG-Related Indices and MASLD Risk

To assess the effects of TyG-related indices, weighted logistic regression analyses were carried out in this study using TyG-related indices as categorical and continuous (per standard deviation (SD) increase) variables. In each weighted logistic regression model, only TyG-BMI correlated significantly with MASLD. This trend consistently persisted across different quartiles of the TyG-BMI, as shown in Table 2.  Table 3 shows positive correlations between TyG-related indices and the risk of MASLD for sex, age, race, hypertension status, and diabetes status. Furthermore, a significant interaction was observed between age, sex, and the risk of TyG-BMI-associated MASLD (*p* < 0.05 for interaction). Stratified analyses by sex and age revealed that the risk of TyG-BMI-associated MASLD was greater in males than in females and in individuals aged 60 years and older than in those under 60 years of age.

**Figure 2:**
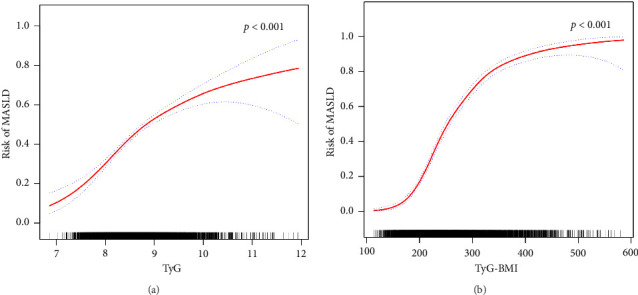
Relationships between TyG (a), TyG-BMI (b), and MASLD risk, depicted by a smooth curve fit (red band) with a 95% confidence interval (blue band). The adjustment strategy is the same as that of the fully adjusted model.

Furthermore, in accordance with Figure 2, the utilization of smooth curve fitting analyses was used to examine the nonlinear correlation between the TyG-related indices and MASLD. The smooth curve demonstrated a favorable connection between these indices and MASLD, and its adjustment strategy mirrored that of the fully adjusted model (both *p* < 0.001).  Figure 2(a) illustrates the alterations in MASLD risk in relation to TyG, with no notable saturation or threshold effect evident. Notably, the analysis shown in Figure 2(b) revealed the variations in MASLD risk in relation to TyG-BMI. Initially, the changes were minimal; however, upon reaching a specific threshold of TyG-BMI, the MASLD risk exhibited a substantial increase, indicating a piecewise linear association. To further evaluate this threshold effect, a two-piecewise logistic regression model was employed. The fitted curve was examined, and the inflection point was determined to be 180.71, as demonstrated in Supporting Table 2. Statistical significance was observed at the inflection point of 180.71 for the LRT conducted on the TyG-BMI (*p* < 0.001), which suggested that the two-piece regression model was suitable for describing the association between TyG-BMI and MASLD risk.

### 3.3. TyG-Related Indices for the Identification of MASLD: AUC, Reclassification, and DCA Curves

**Figure 3:**
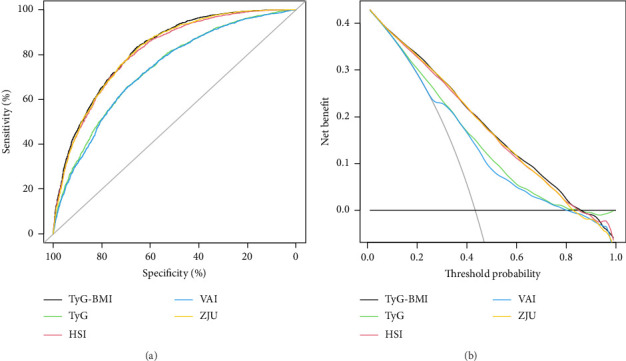
ROC curves for TyG, TyG-BMI, HSI, VAI, and ZJU for predicting MASLD risk, with specificity on the *x*-axis and sensitivity on the *y*-axis (a). Decision curves were used to evaluate the clinical utility of these indices, plotting the threshold probability on the *x*-axis against the net benefits on the *y*-axis, which accounted for true positives minus false positives (b).

The HSI [34], VAI [32], and ZJU [33] are common noninvasive models used to diagnose MASLD. To assess the efficacy of TyG-related indices in identifying MASLD, we conducted a comparative analysis of TyG, TyG-BMI, HSI, VAI, and ZJU in terms of their specificity (SPE) and sensitivity (SEN) in predicting MASLD. The ROC curves of all five models are presented in Figure 3(a), and the performance details are provided in Table 4. Our findings indicated that TyG-BMI exhibited the highest AUC (0.820 (95% CI 0.810–0.831)) among the tested noninvasive scores, surpassing those of HSI (0.811 (0.800–0.822)), VAI (0.731 (0.718–0.744)), ZJU (0.816 (0.805–0.827)), and TyG (0.736 (0.723–0.749)) in predicting the risk of MASLD. Furthermore, the TyG-BMI was significantly different from the other noninvasive scores (all *p* < 0.05) in terms of the AUC. The TyG-BMI had a SPE of 65.3%, SEN of 83.5%, negative predictive value (NPV) of 72.5%, and positive predictive value (PPV) of 62.4%, with a cutoff of 240.153.

Since the clinical meaning of AUC increments is not intuitive, to further evaluate the predictive ability of the TyG-related indices, we calculated the NRI and IDI and compared them with the HSI, VAI, and ZJU. The findings indicated that the NRI and IDI values of the TyG-BMI, compared with those of the TyG-BMI and VAI, were both above zero and demonstrated statistical significance. However, compared with those of HSI and ZJU, the NRI and IDI values of TyG-BMI were also above zero but did not reach statistical significance (as shown in Supporting Table 3). Furthermore, DCA curves were used to assess the clinical efficacy of these indices, revealing that the TyG-BMI provided greater net benefits than other noninvasive indices did within the 0.01–0.85 threshold range, peaking at a net benefit of 0.428, as shown in Figure 3(b).

### 3.4. The Relationship Between the TyG-Related Indices and SLF Risk in MASLD

**Figure 4:**
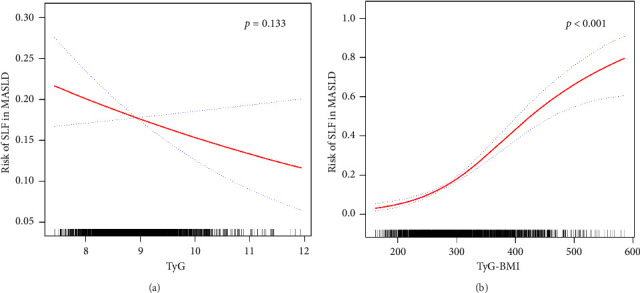
Relationships among TyG (a), TyG-BMI (b), and the risk of SLF in MASLD participants, depicted by a smooth curve fit (red band) and its 95% confidence interval (blue band). The adjustment strategy is the same as that of the fully adjusted model.

Table 5 shows that there was no significant association between the TyG index and SLF in the minimally and fully adjusted models. However, the TyG-BMI was positively correlated with the SLF in all three models (all *p* < 0.05). In further subgroup analyses, only TyG-BMI was positively associated with SLF risk in all subgroups, as presented in Table 6. Interestingly, no interaction was detected between subgroups and TyG-BMI-associated SLF risk. Therefore, analyses of nonlinear relationships are necessary. In this study (Figure 4 and Supporting Table 4), we found a nonlinear association between TyG-BMI and SLF (*p* < 0.001), but no significant saturation or threshold effects were detected (*p* for LRT test = 0.256).

Despite the exploration of nonlinear correlations (as shown in Figure 4 and Supporting Table 4), a significant positive correlation between the TyG-BMI and SLF risk was found. However, the analysis of curve traits and saturation threshold effects indicated no significant nonlinear relationship between them (*p* for LRT test = 0.256). In contrast, in terms of TyG and SLF, there was no significant correlation (*p*=0.133).

### 3.5. Performance of the TyG-Related Indices in Terms of the AUC, Reclassification, and DCA Curve in Predicting SLF in MASLD

**Figure 5:**
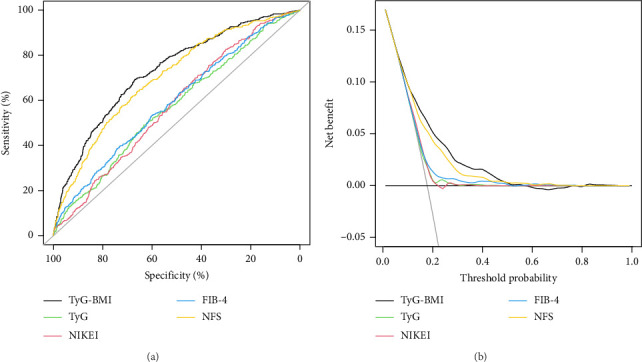
ROC curves for TyG, TyG-BMI, NIKEI, FIB-4, and NFS for predicting the risk of SLF in MASLD patients, with specificity on the *x*-axis and sensitivity on the *y*-axis (a). Decision curves were used to evaluate the clinical utility of these indices, plotting the threshold probability on the *x*-axis against the net benefits on the *y*-axis, which accounted for true positives minus false positives (b).

We compared the ability of TyG-related indices and noninvasive models commonly used to predict liver fibrosis (NIKEI [36], FIB-4 [35], and NFS [37]) in predicting SLF in MASLD. The results, as depicted in Figure 5(a), illustrate the ROC curves for all the models, and the detailed performance results can be found in Table 7. Notably, TyG-BMI exhibited the highest AUC of 0.729 (95% CI 0.703–0.756) for predicting SLF in MASLD, surpassing the AUC values of the NIKEI (0.577 (0.549–0.606)), FIB-4 (0.591 (0.562–0.621)), NFS (0.702 (0.675–0.729)), and TyG (0.572 (0.542–0.602)). With the exception of the NFS, the difference between the TyG-BMI and the AUC of the other noninvasive scores was statistically significant. There was a 67.0% SEN, 69.2% SPE, 31.2% PPV, and 90.5% NPV of the TyG-BMI, with a cutoff of 310.136.

Moreover, compared with NIKEI, FIB-4, and TyG, the NRI and IDI values for TyG-BMI were above zero (as shown Supporting Table 5). There was no significant difference in the NRI or IDI values between the TyG-BMI and NFS. Additionally, as shown in Figure 5(b), TyG-BMI provided a greater net benefit than the other models within the threshold range of approximately 0.01–0.52, reaching a maximum net benefit of 0.170.

## 4. Discussion

IR plays a pivotal role in the pathogenesis of NAFLD, and the hyperinsulinemic-euglycemic clamp method is considered the benchmark for assessing IR [45]. Nevertheless, its impracticality in clinical settings due to its time consumption and complexity has led to the emergence of surrogate indices for diagnosing IR. Notably, the TyG index and TyG-BMI have demonstrated successful application in numerous studies, with favorable SEN and SPE [12, 13]. Thus, they can be used as surrogate markers for IR assessment. Furthermore, TyG has been found to be associated with the risk of NAFLD: Lee et al. [46] reported a significant correlation between TyG and the prevalence of NAFLD, with TyG outperforming HOMA-IR in predicting NAFLD among Korean adults. Similarly, a study conducted in 2016 involving 50 women demonstrated that TyG was the preferred parameter for screening NAFLD patients compared with other existing predictive models [19]. Additionally, a cross-sectional study of 10,761 Chinese individuals in 2017 revealed that the TyG score was a valuable tool for identifying individuals at risk for NAFLD, with an AUC of 0.782, 95% CI of 0.773–0.792, and a cutoff point of 8.5 [20]. Moreover, a population-based cohort study of Japanese people revealed that the TyG index was significantly associated with incident NAFLD [17]. On the other hand, a substantial positive correlation was observed between the TyG-BMI and the risk of NAFLD, surpassing other conventional indicators in its ability to predict NAFLD. In a study conducted by Zhang et al. on nonobese Chinese individuals, adjustments for potential confounding factors revealed that each one SD increase in TyG-BMI yielded an odds ratio of 3.4 (95% CI 3.0–3.9) for NAFLD [47]. Moreover, the aforementioned study reported an AUC of 0.835 (95% CI, 0.824–0.845) for the TyG-BMI, demonstrating superior diagnostic performance compared with TyG, TG, FPG, and BMI in identifying individuals with NAFLD. In their study, Lim demonstrated that, upon accounting for potential confounding variables, the odds ratios (95% CI) for NAFLD in a Korean population were 16.1 (13.4–19.2) for the TyG index and 39.2 (31.6–48.6) for the TyG-BMI. Additionally, the AUCs for each parameter were 0.786 (0.777–0.796) for TyG and 0.837 (0.828–0.846) for TyG-BMI [18]. In 2020, a research study conducted in Iran examined the associations between TyG-related indices and NAFLD in a sample of 184 overweight or obese individuals. The study consistently revealed significant correlations between the TyG-related indices and NAFLD. The standardized ORs for NAFLD were 2.21 for TyG and 2.01 for TyG-BMI, with corresponding AUC values of 0.676 (95% CI: 0.598–0.754) for TyG and 0.675 (95% CI: 0.598–0.752) for TyG-BMI [30]. In 2021, a study involving 14,251 general subjects not only revealed a link between NAFLD and the TyG-BMI but also confirmed that the TyG-BMI had the best ability to discriminate NAFLD [48]. Peng et al. utilized the NHANES database to evaluate the predictive significance of TyG-related indices in MAFLD/NAFLD patients. Their findings revealed that the AUC for the TyG-BMI was 0.867 (95% CI: 0.841–0.889) in relation to the risk of MAFLD and 0.836 (95% CI: 0.808–0.861) in relation to the risk of NAFLD [49]. In addition, several studies have investigated noninvasive scores indicative of IR, demonstrating their correlation with NAFLD and their diagnostic utility for this condition. For instance, Cai et al. examined the association between the metabolic score for IR (METS-IR) and the risk of NAFLD in a cohort of 10,730 Chinese nonobese individuals. Their findings indicated that the AUC values for METS-IR in predicting NAFLD risk over a period of one to four years were 0.784, 0.756, 0.758, and 0.752, respectively. This study is among the limited research efforts that have explored noninvasive scores reflecting IR in relation to the risk of NAFLD development, thereby reinforcing the link between IR and NAFLD [50]. The aforementioned studies have several limitations. First, a number of studies suffer from inadequate sample sizes and a lack of reliability. Second, the majority of studies have relied on ultrasonography as a diagnostic tool for NAFLD/MAFLD, potentially resulting in misclassification due to the limited accuracy of ultrasonography in detecting mild hepatic steatosis [51]. Third, the evaluation of the predictive value of TyG-related indices for NAFLD/MAFLD is restricted to assessing the AUC without further examination from a clinical standpoint, such as the utilization of DCA curves, the NRI, and IDI calculations. Finally, few studies have examined subgroups, and most studies have been conducted in limited populations. Thus, it was unclear whether their findings can be generalized.

On the basis of the findings of this study, a significant association between TyG-related indices and the risk of MASLD in various subgroups was identified. Notably, the TyG-BMI exhibited the most robust correlation with MASLD, as evidenced by a 42.96-fold increased likelihood of MASLD among participants in the highest TyG-BMI quartile compared with those in the lowest quartile, even after adjusting for covariates. In contrast to prior research, our study further employed smoothed curve fitting analysis. The results revealed a nonlinear association between the MASLD score and the TyG-BMI. Encouragingly, a significant threshold effect was observed in the relationship between the MASLD score and the TyG-BMI. The inflection point was determined through the utilization of a smoothed curve fitting graph, revealing that the positive correlation between TyG-BMI and MASLD becomes more pronounced after surpassing a threshold of 180.71. These findings align with the conclusions drawn by Wang et al. [48]. Over the past several decades, numerous clinical diagnostic models have been developed for NAFLD/MAFLD, including HSI [34], ZJU [33], VAI [32], and various other diagnostic nomograms [52–54]. In this study, we selected widely utilized clinical diagnostic models and conducted a comparative analysis with TyG and TyG-BMI. According to the AUC, the TyG-BMI emerged as the most dependable indicator for identifying MASLD in the current study, surpassing TyG, VAI, HSI, and ZJU. In addition, our analysis revealed that the NRI and IDI of TyG-BMI were not significantly different from those of ZJU but were superior to those of TyG, HSI, and VAI. Notably, the TyG-BMI demonstrated the greatest net benefit according to the DCA curve. Consequently, on the basis of these findings, we propose that the TyG-BMI serves as a more valuable indicator for detecting individuals with MASLD and has the potential to replace intricate clinical predictive models. Interestingly, this study also revealed a significant interaction between age, sex, and TyG-BMI-related MASLD risk, suggesting that males as well as those aged 60 years and above have a higher TyG-BMI-related MASLD risk. Exercise intensity tends to be lower in individuals aged 60 years and older than in those under 60 years of age [55]. Physical activity is now acknowledged as a fundamental component in the prevention and management of MASLD. Insufficient physical activity not only increases susceptibility to MASLD but also contributes to metabolic abnormalities in lipids, blood pressure, and glucose levels, thereby increasing the risk of cardiovascular and cerebrovascular diseases, diabetes, and other related metabolic disorders [56]. The findings of a meta-analysis comprising 54 studies indicate that females exhibit a 19% lower likelihood of developing NAFLD than males do [57]. This disparity is attributed to the influence of sex hormones, particularly estrogen, as well as variations in body immunity, adipose distribution, histopathological characteristics, and the intestinal flora between sexes, all of which are implicated in the pathogenesis of MASLD. Consequently, males are more susceptible to MASLD than females are [58].

The link between indicators associated with IR and SLF is more complicated, although IR has the exact opposite effect on lipid metabolism as hepatic fibrosis does. Some studies have explored the link between TyG indices and liver fibrosis risk in NAFLD patients. Guo et al. [16] reported a positive association between TyG and liver fibrosis in Chinese NAFLD patients after adjusting for confounding factors. Furthermore, a study of 5158 Koreans [59] revealed that higher TyG levels worsened NAFLD and liver fibrosis. Similarly, Khamseh et al. suggested that TyG and TyG-BMI could identify NAFLD and liver fibrosis in overweight or obese people, with TyG-BMI also predicting liver fibrosis in 184 overweight or obese adults aged 30–65 years [30]. In contrast to previous findings, our analysis did not reveal an association between the TyG index and SLF after controlling for confounding variables. After adjusting for covariates, a positive association was observed between TyG-BMI and SLF. In further subgroup analyses, only TyG-BMI was positively associated with SLF in different subgroups. Smooth curve fitting revealed a consistent increase in SLF risk with increasing TyG-BMI (*p* < 0.001), without a significant nonlinear relationship. Compared with the NIKEI, FIB-4, and TyG, TyG-BMI demonstrated superior predictive performance for SLF, as evidenced by higher AUC, NRI, IDI, and DCA curve metrics. Nevertheless, there was no statistically significant disparity in the predictive efficacy between the TyG-BMI and NFS for patients with SLF in MASLD. The clinical utility of the NFS is limited by its more complex calculation method than that of the TyG-BMI. In conclusion, despite the unsatisfactory predictive ability of the TyG-BMI for SLF, it still outperforms the NIKEI, FIB-4, and TyG and requires fewer variables than the NFS does. This finding indicates that the TyG-BMI may serve as a viable variable screening option in future predictive model studies.

This study has several noteworthy strengths. First, hepatic steatosis and stiffness were assessed via VCTE, a method known for its heightened sensitivity and accuracy compared with those of ultrasonography [49]. Second, this study successfully demonstrated a nonlinear association between the TyG-BMI and MASLD risk, revealing the existence of a threshold effect and identifying a cutoff point that has significant clinical implications. Finally, this study conducted a comprehensive evaluation of the impact of TyG-related indices on the risk of both MASLD and SLF. In addition to assessing the AUC, we conducted a comparative analysis of the IDI, NRI, and DCA curves, which possess greater relevance to clinical values, in relation to widely employed ROC analysis. Furthermore, it is important to acknowledge several limitations in our research. First, the design of our study was cross-sectional, which precludes the establishment of a causal relationship. Second, in observational studies, certain confounding factors may remain unmeasured or unassessed, leading to the possibility of residual confounding. Finally, the impracticality and invasiveness of conducting liver biopsies on a large sample size prevented us from utilizing this diagnostic gold standard in our study.

## 5. Conclusion

This study identified a statistically significant link between elevated TyG-related indices, namely, TyG and TyG-BMI, and the risk of MASLD in the U.S. population. The TyG-BMI demonstrated significant predictive value for MASLD risk, with an AUC of 0.820 (95% CI 0.810–0.831). Additionally, our study revealed a consistent increase in the risk of SLF in MASLD patients with higher TyG-BMI values, and the TyG-BMI also has good diagnostic value for SLF, with an AUC of 0.729 (95% CI 0.703–0.756). After controlling for confounding variables, our study revealed no association between the TyG score and the SLF. This study underscores the enhanced diagnostic utility of the TyG-BMI index for MASLD and SLF, suggesting it as a potentially simpler and more cost-effective method for identifying individuals at elevated risk for these conditions.

## Figures and Tables

**Table 1:** Weighted baseline characteristics of participants with or without MASLD assessed by VCTE in the NHANES database, 2017–2020.3.

Variables	Non-MASLD (*n* = 3125)	MASLD (*n* = 2395)	*p* value
*Demographic parameters*			
Age (years)	44.14 (0.76)	50.38 (0.71)	< 0.001
Male (%)	44.20 (1.36)	56.31 (1.65)	< 0.001
Race (%)			< 0.001
Non-Hispanic black	11.31 (1.47)	8.37 (1.17)	
Non-Hispanic white	65.11 (2.59)	63.16 (2.55)	
Hispanic	14.81 (1.43)	19.73 (2.04)	
Non-Hispanic Asian	4.96 (0.89)	4.22 (0.71)	
Other races	3.81 (0.39)	4.52 (0.69)	
*Anthropometric parameters*			
BMI (kg/m^2^)	26.68 (0.20)	34.05 (0.27)	< 0.001
WC (cm)	92.18 (0.50)	111.79 (0.62)	< 0.001
*VCTE parameters*			
CAP (dB/m)	219.09 (0.90)	323.70 (1.17)	< 0.001
LSM (kPa)	4.93 (0.08)	7.04 (0.25)	< 0.001
*Serum test*			
PLT (10^6^/L)	245.47 (2.49)	249.69 (2.27)	0.135
TBIL (mg/dL)	0.49 (0.01)	0.46 (0.01)	0.049
ALT (U/L)	18.73 (0.21)	27.06 (0.56)	< 0.001
AST (U/L)	20.03 (0.22)	22.53 (0.28)	< 0.001
GGT (U/L)	22.27 (0.46)	34.58 (0.92)	< 0.001
ALP (U/L)	71.78 (0.96)	78.46 (0.87)	< 0.001
TP (g/L)	71.01 (0.16)	70.96 (0.17)	0.781
ALB (g/L)	41.61 (0.14)	40.84 (0.14)	< 0.001
GLB (g/L)	29.40 (0.15)	30.12 (0.18)	< 0.001
FPG (mg/dL)	92.21 (0.47)	106.69 (1.27)	< 0.001
TG (mg/dL)	110.89 (2.13)	174.39 (3.82)	< 0.001
TC (mg/dL)	184.26 (1.23)	189.23 (2.01)	0.009
HDL (mg/dL)	56.93 (0.52)	47.53 (0.40)	< 0.001
LDL (mg/dL)	105.40 (0.94)	107.17 (1.68)	0.291
UA (mg/dL)	5.01 (0.04)	5.76 (0.05)	< 0.001
CRE (mg/dL)	0.86 (0.00)	0.89 (0.01)	0.011
eGFR (mL/min/m^2^)	97.48 (0.85)	93.15 (0.93)	< 0.001
*Noninvasive indices and models*			
TyG	8.40 (0.02)	8.94 (0.02)	< 0.001
TyG-BMI	224.90 (1.84)	304.60 (2.49)	< 0.001
HSI	35.22 (0.23)	44.78 (0.34)	< 0.001
VAI	1.62 (0.05)	3.03 (0.08)	< 0.001
ZJU	36.91 (0.21)	46.32 (0.29)	< 0.001
*Metabolic diseases*			
Hypertension (%)	20.83 (1.15)	43.86 (1.95)	< 0.001
Diabetes (%)	6.41 (0.67)	25.40 (1.49)	< 0.001
*Lifestyle*			
Smoking (%)			0.005
Never	59.66 (1.61)	56.77 (2.00)	
Former	24.00 (1.22)	30.51 (1.88)	
Current	16.34 (1.37)	12.72 (1.52)	
Alcohol consumption (g/week)	28.22 (1.63)	26.57 (1.40)	0.434

*Note:* Continuous variables are shown as mean (SE), and their *p* value was calculated by weighted linear regression model. Categorical values are shown as % (SE), and its *p* value was calculated by weighted Chi-square test.

Abbreviations: ALB, albumin; ALP, alkaline phosphatase; ALT, alanine aminotransferase; AST, aspartate aminotransferase; BMI, body mass index; CAP, controlled attenuation parameter; CRE, creatinine; eGFR, estimated glomerular filtration rate; FPG, fasting plasma glucose; GGT, γ-glutamyl transpeptidase; GLB, globulin; HDL, high-density lipoprotein cholesterol; LDL, low-density lipoprotein cholesterol; LSM, liver stiffness measurements; MASLD, metabolic dysfunction associated steatotic liver disease; NHANES, National Health and Nutrition Examination Survey; PLT, platelet; SE, standard error of mean; TBIL, total bilirubin; TC, total cholesterol; TG, triglyceride; TP, total protein; UA, uric acid; VCTE, vibration-controlled transient elastography; WC, waist circumference.

**Table 2:** Weighted multivariate analysis of the relationship between TyG-related indices and MASLD.

Variables	Crude model (OR, 95% CI, *p*)	Minimally adjusted model (OR, 95% CI, *p*)	Fully adjusted model (OR, 95% CI, *p*)
Baseline TyG (per SD increase)	2.99 (2.74, 3.26) < 0.001	2.42 (2.21, 2.66) < 0.001	1.85 (1.65, 2.08) < 0.001
Quartiles of TyG			
Q1 (6.87–8.17)	Ref	Ref	Ref
Q2 (8.17–8.57)	2.93 (2.21, 3.89) < 0.001	2.60 (1.90, 3.54) < 0.001	1.94 (1.40, 2.68) 0.010
Q3 (8.57–9.02)	5.69 (4.21, 7.70) < 0.001	4.38 (3.04, 6.30) < 0.001	2.65 (1.78, 3.95) 0.005
Q4 (9.02–12.34)	13.29 (10.67, 16.56) < 0.001	7.67 (5.95, 9.87) < 0.001	3.77 (2.80, 5.08) < 0.001
*p* for trend	< 0.001	< 0.001	< 0.001
Baseline TyG-BMI (per SD increase)	5.32 (4.47, 6.33) < 0.001	4.98 (4.12, 6.03) < 0.001	4.44 (3.64, 5.42) < 0.001
Quartiles of TyG-BMI			
Q1 (114.08–208.93)	Ref	Ref	Ref
Q2 (208.93–249.62)	8.29 (5.63, 12.20) < 0.001	6.68 (4.39, 10.16) < 0.001	5.99 (3.88, 9.26) < 0.001
Q3 (249.62–300.63)	22.83 (15.06, 34.62) < 0.001	17.22 (10.56, 28.09) < 0.001	14.54 (8.56, 24.71) < 0.001
Q4 (300.63–666.79)	77.75 (48.56, 124.48) < 0.001	57.13 (34.44, 94.77) < 0.001	43.96 (25.28, 76.42) < 0.001
*p* for trend	< 0.001	< 0.001	< 0.001

*Note:* Crude model adjusted for none. Minimally adjusted model adjusted for age, sex, race, alcohol consumption, LSM, diabetes, and hypertension. Fully adjusted model adjusted for age, sex, race, alcohol consumption, LSM, diabetes, hypertension, ALT, AST, ALP, ALB, GGT, HDL, and eGFR.

**Table 3:** Weighted stratified associations between TyG-related indices and MASLD by age, sex, race, hypertension, and diabetes.

Subgroup	TyG		TyG-BMI	
	Adjusted OR (95% CI)	*p* for interaction	Adjusted OR (95% CI)	*p* for interaction
Age (years)		0.309		0.009
< 60	2.00 (1.75, 2.28)		4.76 (3.68, 6.14)	
≥ 60	1.76 (1.42, 2.18)		3.42 (2.74, 4.28)	
Sex		0.735		< 0.001
Female	1.80 (1.46, 2.23)		3.45 (2.74, 4.34)	
Male	1.88 (1.67, 2.13)		7.04 (5.61, 8.84)	
Race		0.057		0.559
Non-Hispanic black	1.51 (1.28, 1.79)		3.48 (2.87, 4.22)	
Non-Hispanic white	2.00 (1.69, 2.36)		4.66 (3.40, 6.38)	
Hispanic	1.67 (1.42, 1.97)		4.81 (3.46, 6.67)	
Non-Hispanic Asian	2.21 (1.62, 3.03)		5.76 (2.54, 13.07)	
Other races	1.38 (0.90, 2.11)		3.23 (2.09, 5.01)	
Hypertension		0.788		0.508
No	1.83 (1.60, 2.09)		4.62 (3.54, 6.02)	
Yes	1.90 (1.51, 2.38)		4.09 (3.18, 5.27)	
Diabetes		0.524		0.386
No	1.97 (1.70, 2.27)		4.57 (3.62, 5.76)	
Yes	1.70 (1.36, 2.14)		3.74 (2.60, 5.38)	

*Note:* Adjust for age, sex, race, alcohol consumption, LSM, diabetes, hypertension, ALT, AST, ALP, ALB, GGT, HDL, and eGFR. In the subgroup analysis, models were not adjusted for their own stratification variable.

**Table 4:** Performance assessment of the TyG, TyG-BMI, HSI, VAI, and ZJU for the prediction of MASLD.

Variables	AUC (95% CI)	SEN (95% CI)	SPE (95% CI)	PPV (95% CI)	NPV (95% CI)	Cutoff value
TyG	0.736 (0.723–0.749)	0.654 (0.635–0.673)	0.697 (0.681–0.713)	0.624 (0.605–0.643)	0.725 (0.709–0.741)	8.638
TyG-BMI	0.820 (0.810–0.831)	0.835 (0.821–0.850)	0.653 (0.637–0.670)	0.649 (0.632–0.666)	0.838 (0.824–0.853)	240.153
HSI	0.811 (0.800–0.822)	0.772 (0.756–0.789)	0.705 (0.689–0.721)	0.667 (0.650–0.685)	0.802 (0.787–0.817)	38.285
VAI	0.731 (0.718–0.744)	0.657 (0.638–0.676)	0.693 (0.677–0.709)	0.621 (0.602–0.640)	0.725 (0.709–0.741)	1.700
ZJU	0.816 (0.805–0.827)	0.827 (0.812–0.842)	0.655 (0.638–0.671)	0.647 (0.630–0.664)	0.831 (0.817–0.846)	38.847

Abbreviations: AUC, area under the receiver operating characteristic curve; NPV, negative predictive value; PPV, positive predictive value; SEN, sensitivity; SPE, specificity.

**Table 5:** Multivariate analysis of the relationship between the TyG-related indices and SLF in MASLD.

Variable	Crude model (OR, 95% CI, *p*)	Minimally adjusted model (OR, 95% CI, *p*)	Fully adjusted model (OR, 95% CI, *p*)
Baseline TyG (per SD increase)	1.37 (1.14, 1.64) 0.002	1.11 (0.93, 1.32) 0.277	0.88 (0.72, 1.08) 0.263
Quartiles of TyG			
Q1 (7.43–8.48)	Ref	Ref	Ref
Q2 (8.48–8.87)	1.88 (1.01, 3.50) 0.050	1.85 (1.02, 3.38) 0.047	1.46 (0.73, 2.91) 0.321
Q3 (8.87–9.29)	2.04 (1.17, 3.56) 0.020	1.72 (0.99, 2.98) 0.076	1.16 (0.59, 2.28) 0.676
Q4 (9.29–12.34)	3.18 (1.96, 5.17) < 0.001	2.05 (1.34, 3.15) 0.006	1.11 (0.60, 2.06) 0.747
*p* for trend	< 0.001	0.070	0.587
Baseline TyG-BMI (per SD increase)	2.51 (2.21, 2.85) < 0.001	2.76 (2.52, 3.02) < 0.001	2.48 (2.15, 2.87) < 0.001
Quartiles of TyG-BMI			
Q1 (162.00–252.25)	Ref	Ref	Ref
Q2 (252.25–292.22)	2.19 (0.62, 7.71) 0.234	1.93 (0.56, 6.61) 0.316	2.14 (0.54, 8.54) 0.318
Q3 (292.22–337.89)	4.77 (1.66, 13.68) 0.008	3.99 (1.44, 11.06) 0.020	4.25 (1.40, 12.88) 0.038
Q4 (337.89–665.05)	17.68 (5.70, 54.83) < 0.001	15.39 (5.15, 46.05) < 0.001	13.60 (1.99, 3.75) 0.003
*p* for trend	< 0.001	< 0.001	< 0.001

*Note:* Crude model adjusted for none. Minimally adjusted model adjusted for age, sex, race, alcohol consumption, diabetes, and hypertension. Fully adjusted model adjusted for age, sex, race, alcohol consumption, diabetes, hypertension, ALT, AST, ALP, GGT, HDL, and CAP.

**Table 6:** Weighted stratified associations between TyG-related indices and SLF in MASLD by age, sex, race, hypertension, and diabetes.

Subgroup	TyG		TyG-BMI	
	Adjusted OR (95% CI)	*p* for interaction	Adjusted OR (95% CI)	*p* for interaction
Age (years)		0.708		0.068
< 60	0.87 (0.73, 1.03)		2.52 (2.13, 2.99)	
≥ 60	0.92 (0.64, 1.33)		2.10 (1.77, 2.49)	
Sex		0.063		0.273
Female	0.71 (0.53, 0.97)		2.19 (1.64, 2.92)	
Male	0.97 (0.77, 1.21)		2.81 (1.63, 4.85)	
Race		0.214		0.693
Non-Hispanic black	0.79 (0.53, 1.20)		2.79 (2.18, 3.56)	
Non-Hispanic white	0.82 (0.64, 1.06)		2.44 (2.03, 2.93)	
Hispanic	0.94 (0.72, 1.22)		2.38 (1.77, 3.18)	
Non-Hispanic Asian	0.81 (0.55, 1.19)		1.99 (1.20, 3.31)	
Other races	2.14 (0.85, 5.39)		3.28 (1.38, 7.81)	
Hypertension		0.805		0.459
No	0.90 (0.71, 1.15)		2.63 (2.02, 3.42)	
Yes	0.86 (0.64, 1.17)		2.34 (2.02, 2.71)	
Diabetes		0.188		0.278
No	0.79 (0.60, 1.04)		2.66 (2.11, 3.37)	
Yes	0.96 (0.76, 1.20)		2.17 (1.76, 2.68)	

*Note:* Adjust for age, sex, race, alcohol consumption, diabetes, hypertension, ALT, AST, ALP, GGT, HDL, and CAP. In the subgroup analysis, models were not adjusted for their own stratification variable.

**Table 7:** Performance assessment of the TyG, TyG-BMI, NIKEI, FIB-4, and NFS for the prediction of SLF in MASLD.

Variables	AUC (95% CI)	SEN (95% CI)	SPE (95% CI)	PPV (95% CI)	NPV (95% CI)	Cutoff value
TyG	0.572 (0.542–0.602)	0.491 (0.443–0.538)	0.634 (0.613–0.655)	0.225 (0.198–0.252)	0.852 (0.834–0.870)	9.027
TyG-BMI	0.729 (0.703–0.756)	0.692 (0.649–0.736)	0.670 (0.650–0.691)	0.312 (0.283–0.342)	0.905 (0.886–0.923)	310.136
NIKEI	0.577 (0.549–0.606)	0.826 (0.790–0.862)	0.301 (0.280–0.321)	0.204 (0.185–0.223)	0.858 (0.837–0.878)	10.625
FIB-4	0.591 (0.562–0.621)	0.533 (0.485–0.580)	0.602 (0.580–0.623)	0.225 (0.199–0.250)	0.856 (0.838–0.875)	1.030
NFS	0.702 (0.675–0.729)	0.641 (0.595–0.686)	0.661 (0.640–0.682)	0.290 (0.261–0.319)	0.895 (0.879–0.911)	−0.625

Abbreviations: AUC, area under the receiver operating characteristic curve; NPV, negative predictive value; PPV, positive predictive value; SEN, sensitivity; SPE, specificity.

## Data Availability

The data that support the findings of this study are openly available in the NHANES at https://www.cdc.gov/nchs/nhanes/index.htm.

## Supporting Information

To avoid excessive word counts in the main manuscript as well as more figures than required by the journal, we added a document called “Supporting Information” to the manuscript at the time of submission. This Supporting Information is divided into five sections: (1) definitions of the variables used in the study, (2) formulas for calculating noninvasive predictive scores, (3) definitions of MASLD, (4) references cited in the Supporting Information, and (5) Supporting tables. The tables include: Table S1, which presents weighted baseline characteristics of participants with or without SLF in the NHANES; Table S2, which analyzes the threshold and saturation effects between TyG-BMI and MASLD; Table S3, which assesses the performance of TyG, TyG-BMI, HSI, VAI, and ZJU in predicting MASLD; Table S4, which examines the threshold and saturation effects between TyG-BMI and SLF; and Table S5, which evaluates the performance of TyG, TyG-BMI, NIKEI, FIB-4, and NFS in predicting SLF in MASLD.

## References

[1] Younossi Z., Anstee Q. M., Marietti M. (2018). Global Burden of NAFLD and NASH: Trends, Predictions, Risk Factors and Prevention. *Nature Reviews Gastroenterology and Hepatology*.

[2] Younossi Z. M., Stepanova M., Younossi Y. (2020). Epidemiology of Chronic Liver Diseases in the USA in the Past Three Decades. *Gut*.

[3] Eslam M., Sanyal A. J., George J. (2020). MAFLD: A Consensus-Driven Proposed Nomenclature for Metabolic Associated Fatty Liver Disease. *Gastroenterology*.

[4] Younossi Z. M., Rinella M. E., Sanyal A. J. (2021). From NAFLD to MAFLD: Implications of a Premature Change in Terminology. *Hepatology*.

[5] Rinella M. E., Lazarus J. V., Ratziu V. (2023). A Multi-Society Delphi Consensus Statement on New Fatty Liver Disease Nomenclature. *Journal of Hepatology*.

[6] Angulo P., Kleiner D. E., Dam-Larsen S. (2015). Liver Fibrosis, But No Other Histologic Features, Is Associated With Long-Term Outcomes of Patients With Nonalcoholic Fatty Liver Disease. *Gastroenterology*.

[7] Hagström H., Nasr P., Ekstedt M. (2017). Fibrosis Stage But Not NASH Predicts Mortality and Time to Development of Severe Liver Disease in Biopsy-Proven NAFLD. *Journal of Hepatology*.

[8] Taylor R. S., Taylor R. J., Bayliss S. (2020). Association Between Fibrosis Stage and Outcomes of Patients With Nonalcoholic Fatty Liver Disease: A Systematic Review and Meta-Analysis. *Gastroenterology*.

[9] (2020). The Global, Regional, and National Burden of Cirrhosis by Cause in 195 Countries and Territories, 1990–2017: A Systematic Analysis for the Global Burden of Disease Study 2017. *The Lancet Gastroenterology and Hepatology*.

[10] Khan R. S., Bril F., Cusi K., Newsome P. N. (2019). Modulation of Insulin Resistance in Nonalcoholic Fatty Liver Disease.

[11] Wang X. J., Malhi H. (2018). Nonalcoholic Fatty Liver Disease. *Annals of Internal Medicine*.

[12] Guerrero-Romero F., Simental-Mendía L. E., González-Ortiz M. (2010). The Product of Triglycerides and Glucose, a Simple Measure of Insulin Sensitivity. Comparison With the Euglycemic-Hyperinsulinemic Clamp. *Journal of Clinical Endocrinology and Metabolism*.

[13] Simental-Mendía L. E., Rodríguez-Morán M., Guerrero-Romero F. (2008). The Product of Fasting Glucose and Triglycerides as Surrogate for Identifying Insulin Resistance in Apparently Healthy Subjects. *Metabolic Syndrome and Related Disorders*.

[14] Lim J., Kim J., Koo S. H., Kwon G. C. (2019). Comparison of Triglyceride Glucose Index, and Related Parameters to Predict Insulin Resistance in Korean Adults: An Analysis of the 2007–2010 Korean National Health and Nutrition Examination Survey. *PLoS One*.

[15] Er L. K., Wu S., Chou H. H. (2016). Triglyceride Glucose-Body Mass Index Is a Simple and Clinically Useful Surrogate Marker for Insulin Resistance in Nondiabetic Individuals. *PLoS One*.

[16] Guo W., Lu J., Qin P. (2020). The Triglyceride-Glucose Index Is Associated With the Severity of Hepatic Steatosis and the Presence of Liver Fibrosis in Non-Alcoholic Fatty Liver Disease: A Cross-Sectional Study in Chinese Adults. *Lipids in Health and Disease*.

[17] Kitae A., Hashimoto Y., Hamaguchi M., Obora A., Kojima T., Fukui M. (2019). The Triglyceride and Glucose Index Is a Predictor of Incident Nonalcoholic Fatty Liver Disease: A Population-Based Cohort Study. *Canadian Journal of Gastroenterology and Hepatology*.

[18] Lim J. (2019). Validation of Fatty Liver Index in a Healthy Korean Population and Its Comparison With Triglyceride Glucose Index and Its Related Parameters. *Clinica Chimica Acta*.

[19] Simental-Mendía L. E., Simental-Mendía E., Rodríguez-Hernández H., Rodríguez-Morán M., Guerrero-Romero F. (2016). The Product of Triglycerides and Glucose as Biomarker for Screening Simple Steatosis and NASH in Asymptomatic Women. *Annals of Hepatology*.

[20] Zhang S., Du T., Zhang J. (2017). The Triglyceride and Glucose Index (TyG) Is an Effective Biomarker to Identify Nonalcoholic Fatty Liver Disease. *Lipids in Health and Disease*.

[21] Chen J., Mao X., Deng M., Luo G. (2023). Validation of Nonalcoholic Fatty Liver Disease (NAFLD) Related Steatosis Indices in Metabolic Associated Fatty Liver Disease (MAFLD) and Comparison of the Diagnostic Accuracy Between NAFLD and MAFLD. *European Journal of Gastroenterology and Hepatology*.

[22] Zou H., Ma X., Zhang F., Xie Y. (2023). Comparison of the Diagnostic Performance of Twelve Noninvasive Scores of Metabolic Dysfunction-Associated Fatty Liver Disease. *Lipids in Health and Disease*.

[23] Bae J. C., Beste L. A., Utzschneider K. M. (2022). The Impact of Insulin Resistance on Hepatic Fibrosis Among United States Adults With Non-Alcoholic Fatty Liver Disease: NHANES 2017 to 2018. *Endocrinology and metabolism (Seoul, Korea)*.

[24] Ballestri S., Nascimbeni F., Romagnoli D., Lonardo A. (2016). The Independent Predictors of Non-Alcoholic Steatohepatitis and Its Individual Histological Features: Insulin Resistance, Serum Uric Acid, Metabolic Syndrome, Alanine Aminotransferase and Serum Total Cholesterol Are a Clue to Pathogenesis and Candidate Targets for Treatment. *Hepatology Research: The Official Journal of the Japan Society of Hepatology*.

[25] Fujii H., Imajo K., Yoneda M. (2019). HOMA-IR: An Independent Predictor of Advanced Liver Fibrosis in Nondiabetic Non-Alcoholic Fatty Liver Disease. *Journal of Gastroenterology and Hepatology*.

[26] Kessoku T., Yoneda M., Sumida Y. (2015). Insulin Resistance Correlated With the Severity of Liver Histology in Japanese NAFLD Patients: A Multicenter Retrospective Study. *Journal of Clinical Gastroenterology*.

[27] Chen Z., Yu R., Xiong Y., Du F., Zhu S. (2017). A Vicious Circle Between Insulin Resistance and Inflammation in Nonalcoholic Fatty Liver Disease. *Lipids in Health and Disease*.

[28] Finck B. N. (2018). Targeting Metabolism, Insulin Resistance, and Diabetes to Treat Nonalcoholic Steatohepatitis. *Diabetes*.

[29] Khan R. S., Bril F., Cusi K., Newsome P. N. (2019). Modulation of Insulin Resistance in Nonalcoholic Fatty Liver Disease. *Hepatology*.

[30] Khamseh M. E., Malek M., Abbasi R., Taheri H., Lahouti M., Alaei-Shahmiri F. (2021). Triglyceride Glucose Index and Related Parameters (Triglyceride Glucose-Body Mass Index and Triglyceride Glucose-Waist Circumference) Identify Nonalcoholic Fatty Liver and Liver Fibrosis in Individuals With Overweight/Obesity. *Metabolic Syndrome and Related Disorders*.

[31] Cuschieri S. (2019). The STROBE Guidelines. *Saudi Journal of Anaesthesia*.

[32] Amato M. C., Giordano C., Galia M. (2010). Visceral Adiposity Index: A Reliable Indicator of Visceral Fat Function Associated With Cardiometabolic Risk. *Diabetes Care*.

[33] Wang J., Xu C., Xun Y. (2015). ZJU Index: A Novel Model for Predicting Nonalcoholic Fatty Liver Disease in a Chinese Population. *Scientific Reports*.

[34] Lee J., Kim D., Kim H. (2010). Hepatic Steatosis Index: A Simple Screening Tool Reflecting Nonalcoholic Fatty Liver Disease. *Digestive and Liver Disease*.

[35] Sterling R. K., Lissen E., Clumeck N. (2006). Development of a Simple Noninvasive Index to Predict Significant Fibrosis in Patients With HIV/HCV Coinfection. *Hepatology*.

[36] Demir M., Lang S., Schlattjan M. (2013). NIKEI: A New Inexpensive and Non-Invasive Scoring System to Exclude Advanced Fibrosis in Patients With NAFLD. *PLoS One*.

[37] Angulo P., Hui J. M., Marchesini G. (2007). The NAFLD Fibrosis Score: A Noninvasive System That Identifies Liver Fibrosis in Patients With NAFLD. *Hepatology*.

[38] Eddowes P. J., Sasso M., Allison M. (2019). Accuracy of FibroScan Controlled Attenuation Parameter and Liver Stiffness Measurement in Assessing Steatosis and Fibrosis in Patients With Nonalcoholic Fatty Liver Disease. *Gastroenterology*.

[39] Akinbami L. J., Chen T. C., Davy O. (2022). National Health and Nutrition Examination Survey, 2017-March 2020 Prepandemic File: Sample Design, Estimation, and Analytic Guidelines. *Vital and Health Statistics—Series 1: Programs and Collection Procedures*.

[40] Lin L., Chen C. Z., Yu X. D. (2013). The Analysis of Threshold Effect Using Empower Stats Software. *Chinese Journal of Epidemiology*.

[41] DeLong E. R., DeLong D. M., Clarke-Pearson D. L. (1988). Comparing the Areas Under Two or More Correlated Receiver Operating Characteristic Curves: A Nonparametric Approach. *Biometrics*.

[42] Kerr K. F., McClelland R. L., Brown E. R., Lumley T. (2011). Evaluating the Incremental Value of New Biomarkers With Integrated Discrimination Improvement. *American Journal of Epidemiology*.

[43] Pencina M. J., D’Agostino R. B. S., D’Agostino R. B., Vasan R. S. (2008). Evaluating the Added Predictive Ability of a New Marker: From Area Under the ROC Curve to Reclassification and Beyond. *Statistics in Medicine*.

[44] Van Calster B., Wynants L., Verbeek J. F. M. (2018). Reporting and Interpreting Decision Curve Analysis: A Guide for Investigators. *European Urology*.

[45] DeFronzo R. A., Tobin J. D., Andres R. (1979). Glucose Clamp Technique: A Method for Quantifying Insulin Secretion and Resistance. *American Journal of Physiology*.

[46] Lee S. B., Kim M. K., Kang S. (2019). Triglyceride Glucose Index Is Superior to the Homeostasis Model Assessment of Insulin Resistance for Predicting Nonalcoholic Fatty Liver Disease in Korean Adults. *Endocrinology and metabolism (Seoul, Korea)*.

[47] Zhang S., Du T., Li M. (2017). Triglyceride Glucose-Body Mass Index Is Effective in Identifying Nonalcoholic Fatty Liver Disease in Nonobese Subjects. *Medicine*.

[48] Wang R., Dai L., Zhong Y., Xie G. (2021). Usefulness of the Triglyceride Glucose-Body Mass Index in Evaluating Nonalcoholic Fatty Liver Disease: Insights From a General Population. *Lipids in Health and Disease*.

[49] Peng H., Pan L., Ran S. (2023). Prediction of MAFLD and NAFLD Using Different Screening Indexes: A Cross-Sectional Study in U.S. Adults. *Frontiers in Endocrinology*.

[50] Cai X., Gao J., Hu J. (2022). Dose-Response Associations of Metabolic Score for Insulin Resistance Index With Nonalcoholic Fatty Liver Disease Among a Nonobese Chinese Population: Retrospective Evidence From a Population-Based Cohort Study. *Disease Markers*.

[51] Li Q., Dhyani M., Grajo J. R., Sirlin C., Samir A. E. (2018). Current Status of Imaging in Nonalcoholic Fatty Liver Disease. *World Journal of Hepatology*.

[52] Ji L., Cai X., Bai Y., Li T. (2021). Application of a Novel Prediction Model for Predicting 2-Year Risk of Non-Alcoholic Fatty Liver Disease in the Non-Obese Population With Normal Blood Lipid Levels: A Large Prospective Cohort Study From China. *International Journal of General Medicine*.

[53] Cai X., Aierken X., Ahmat A. (2020). A Nomogram Model Based on Noninvasive Bioindicators to Predict 3-Year Risk of Nonalcoholic Fatty Liver in Nonobese Mainland Chinese: A Prospective Cohort Study. *BioMed Research International*.

[54] Zou H., Zhao F., Lv X., Ma X., Xie Y. (2022). Development and Validation of a New Nomogram to Screen for MAFLD. *Lipids in Health and Disease*.

[55] Golabi P., Locklear C. T., Austin P. (2016). Effectiveness of Exercise in Hepatic Fat Mobilization in Non-Alcoholic Fatty Liver Disease: Systematic Review. *World Journal of Gastroenterology*.

[56] Keating S. E., George J., Johnson N. A. (2015). The Benefits of Exercise for Patients With Non-Alcoholic Fatty Liver Disease. *Expert Review of Gastroenterology and Hepatology*.

[57] Balakrishnan M., Patel P., Dunn-Valadez S. (2021). Women Have a Lower Risk of Nonalcoholic Fatty Liver Disease But a Higher Risk of Progression vs Men: A Systematic Review and Meta-Analysis. *Journal of the American Gastroenterological Association*.

[58] Lonardo A., Nascimbeni F., Ballestri S. (2019). Sex Differences in Nonalcoholic Fatty Liver Disease: State of the Art and Identification of Research Gaps. *Hepatology*.

[59] Kim M. K., Kim J. H., Park K. (2018). Relationship Between the Triglyceride Glucose Index and the Presence and Fibrosis of Nonalcoholic Fatty Liver Disease in Korean Adults. *Diabetes*.

