# Supplementary material for: The Value of TyG-Related Indices in Evaluating MASLD and Significant Liver Fibrosis in MASLD
Source: Can J Gastroenterol Hepatol. 2025 Mar 13;2025:5871321. doi: 10.1155/cjgh/5871321 (PMC11925628; doi:10.1155/cjgh/5871321)
Supplement: Supporting Information — Additional supporting information can be found online in the Supporting Information section. [file 5871321.f1.docx]

**The value of TyG-related indices in evaluating MASLD and significant liver fibrosis in MASLD**

**Supplementary material**

1. **Laboratory measurement and clinical data…………………………………………………………...….….2**
2. **Formula for calculating noninvasive predictive scores……………………………………………….……..2**
3. **The definition of MASLD…………………………………………………………………………………..….3**
4. **Referrences………………………………………………………………………………….……………...…..3**
5. **Supplement tables…………………………………………………………………………………….....….….5**

**Laboratory measurement and clinical data**

The following variables were obtained from the original National Health and Nutrition Examination Survey (NHANES) database: demographic parameters (age, sex, and race), anthropometric parameters (waist circumference (WC), body mass index (BMI)), lifestyles (smoking, alcohol consumption), vibration-controlled transient elastography (VCTE) parameters (liver stiffness measurements (LSM) and controlled attenuation parameter (CAP)), comorbidities (hypertension and diabetes), and biomarkers such as fasting insulin (FINS), fasting plasma glucose (FPG), triglyceride (TG), alanine aminotransferase (ALT), aspartate aminotransferase (AST), alkaline phosphatase (ALP), total bilirubin (TBIL), γ-glutamyl transpeptidase (GGT), total protein (TP), albumin (ALB), and globulin (GLB), total lipoprotein cholesterol (TC), high-density lipoprotein cholesterol (HDL), low-density lipoprotein cholesterol (LDL), uric acid (UA), creatinine (CRE), and estimate glomerular filtration rate (eGFR).

We categorized race into five groups (Hispanic, non-Hispanic Black, non-Hispanic White, non-Hispanic Asian, and other races). Besides, we categorized smoking into three groups (never: smoked <100 cigarettes in a lifetime, former: smoked ≥100 cigarettes in a lifetime and smoke not at all now, and current: smoked ≥100 cigarettes in a lifetime and smoke some days or every day) [1]. The calculation of the average weekly alcohol consumption was determined by considering the frequency of drinking days within the previous year and the mean quantity of alcohol consumed on those specific days (ALQ121 and ALQ130) [2]. Alcohol intake in the NHANES Alcohol Questionnaire is measured in standard drinks, which we converted to grams based on the conversion standard of 1 standard drink =14 grams [3]. Excessive alcohol intake was defined as drinking more than 210g of alcohol per week for men and 140g for women [4]. A history of amiodarone, methotrexate, tamoxifen, aspirin, ibuprofen, entecavir, protease inhibitors, valproic acid, carbamazepine, fluorouracil, irinotecan, and glucocorticosteroids was defined as a history of medication use that causes hepatic steatosis, according to MASLD-related guidelines [5]. Viral hepatitis in the NHANES cohort was defined as the presence of hepatitis C virus infection, indicated by a positive test for viral RNA and/or antibodies, as well as hepatitis B virus infection, indicated by a positive test for surface antigen [6]. The diagnostic criteria for diabetes were glycohemoglobin (HbA1c) > 6.5% or random plasma glucose ≥11.1 mmol/L or FPG ≥7.0 mmol/L or two-hour oral glucose tolerance test (OGTT) plasma glucose ≥11.1 mmol/L or under anti-diabetes therapy, or self-reported diabetes [7]. The definition of hypertension was based on systolic blood pressure (SBP) greater than 140 mmHg or diastolic blood pressure (DBP) greater than 90 mmHg, self-reported hypertension, or under antihypertension treatment [8].

**Formula for calculating noninvasive predictive scores**

The TyG index was calculated using the formula Ln[TG (mg/dL) × FPG (mg/dL) / 2] [9], and the TyG-BMI index was deﬁned as TyG × BMI [10]. The equations for HSI [11], VAI [12], and ZJU [13] were as follows: HSI = 8 × [ALT (U/L) / AST (U/L)] + BMI (kg/m^2^) (+ 2, if diabetes; + 2, if female); VAI = [WC (cm) / (39.68 + 1.88 × BMI (kg/m^2^))] × [TG (mmol/L) / 1.03] × [1.31 / HDL (mmol/L)], for male; [WC (cm) / (36.58 + 1.89 × BMI (kg/m^2^))] × (TG (mmol/L) / 0.81) × (1.52 / HDL (mmol/L)), for female; and ZJU = BMI (kg/m^2^) + FPG (mmol/L) + TG (mmol/L) + 3 × [ALT (U/L) / AST (U/L)] (+ 2, if female). The equations for NIKEI [14], FIB-4 [15], and NFS [16] were as follows: NIKEI = -24.214 + 0.225 × age (years) + AST (U/L) + 5.044 × [AST (U/L) / ALT (U/L)] + 3.631 × TBIL (mg/dL); FIB-4 = [Age (years) × AST (U/L)] / [(PLT (10^9^/L) × $\sqrt{ALT (U/L)}$]; and NFS = -1.675 + 0.037 × Age (years) + 0.094 × BMI (kg/m^2^) + 1.13 × diabetes (yes = 1, no = 0) + 0.99 × [AST (U/L)/ALT (U/L)] - 0.013 × PLT (10^9^/L) - 0.66 × ALB (g/dL).

**The definition of MASLD**

MASLD was defined as the presence of hepatic steatosis with one or more of the following [17]:

1) BMI ≥25 kg/m^2^ (≥23 kg/m^2^ for Asia) OR WC >94 cm (for male), 80 cm (for female) OR ethnicity adjusted;

2) FPG ≥5.6 mmol/L OR two-hour OGTT plasma glucose ≥7.8 mmol/L OR HbA1c ≥5.7% OR type 2 diabetes OR treatment for type 2 diabetes;

3) Blood pressure ≥130/85 mmHg OR specific antihypertensive drug treatment;

4) TG ≥1.70 mmol/L OR lipid lowering treatment;

5) HDL ≤1.0 mmol/L for men OR ≤1.3 mmol/L for women OR lipid lowering treatment.

Patients with excessive alcohol consumption and other causes of hepatic steatosis (viral hepatitis and history of taking medications that may cause hepatic steatosis) were excluded, and the diagnostic criteria for heavy excessive consumption were as described above.

**References**

1. Ruan Z, Lu T, Chen Y, Yuan M, Yu H, Liu R, Xie X: **Association Between Psoriasis and Nonalcoholic Fatty Liver Disease Among Outpatient US Adults**. *JAMA Dermatol* 2022, **158**(7):745-753.

2. **Alcohol Use Questionare** [<https://wwwn.cdc.gov/Nchs/Nhanes/2017-2018/P_ALQ.htm#ALQ121>]

3. **What Is A Standard Drink?** [<https://www.niaaa.nih.gov/alcohols-effects-health/overview-alcohol-consumption/what-standard-drink>]

4. Rinella ME, Lazarus JV, Ratziu V, Francque SM, Sanyal AJ, Kanwal F, Romero D, Abdelmalek MF, Anstee QM, Arab JP *et al*: **A multi-society Delphi consensus statement on new fatty liver disease nomenclature**. *J Hepatol* 2023.

5. **EASL-EASD-EASO Clinical Practice Guidelines on the management of metabolic dysfunction-associated steatotic liver disease (MASLD)**. *Journal of hepatology* 2024, **81**(3):492-542.

6. Younossi ZM, Stepanova M, Afendy M, Fang Y, Younossi Y, Mir H, Srishord M: **Changes in the prevalence of the most common causes of chronic liver diseases in the United States from 1988 to 2008**. *Clinical gastroenterology and hepatology : the official clinical practice journal of the American Gastroenterological Association* 2011, **9**(6):524-530.e521; quiz e560.

7. American Diabetes A: **Classification and Diagnosis of Diabetes: Standards of Medical Care in Diabetes—2020**. *Diabetes Care* 2019, **43**(Supplement_1):S14-S31.

8. Williams B, Mancia G, Spiering W, Agabiti Rosei E, Azizi M, Burnier M, Clement DL, Coca A, de Simone G, Dominiczak A *et al*: **2018 ESC/ESH Guidelines for the management of arterial hypertension**. *European heart journal* 2018, **39**(33):3021-3104.

9. Simental-Mendía LE, Rodríguez-Morán M, Guerrero-Romero F: **The Product of Fasting Glucose and Triglycerides As Surrogate for Identifying Insulin Resistance in Apparently Healthy Subjects**. *Metabolic Syndrome and Related Disorders* 2008, **6**(4):299-304.

10. Lim J, Kim J, Koo SH, Kwon GC: **Comparison of triglyceride glucose index, and related parameters to predict insulin resistance in Korean adults: An analysis of the 2007-2010 Korean National Health and Nutrition Examination Survey**. *PloS one* 2019, **14**(3):e0212963.

11. Lee J, Kim D, Kim H, Lee C, Yang J, Kim W, Kim Y, Yoon J, Cho S, Sung M *et al*: **Hepatic steatosis index: a simple screening tool reflecting nonalcoholic fatty liver disease**. *Digestive and Liver Disease* 2010, **42**(7):503-508.

12. Amato MC, Giordano C, Galia M, Criscimanna A, Vitabile S, Midiri M, Galluzzo A, Group ftAS: **Visceral Adiposity Index: A reliable indicator of visceral fat function associated with cardiometabolic risk**. *Diabetes Care* 2010, **33**(4):920-922.

13. Wang J, Xu C, Xun Y, Lu Z, Shi J, Yu C, Li YJ: **ZJU index: a novel model for predicting nonalcoholic fatty liver disease in a Chinese population**. *Scientific reports* 2015, **5**:16494.

14. Demir M, Lang S, Schlattjan M, Drebber U, Wedemeyer I, Nierhoff D, Kaul I, Sowa J, Canbay A, Töx U *et al*: **NIKEI: a new inexpensive and non-invasive scoring system to exclude advanced fibrosis in patients with NAFLD**. *PloS one* 2013, **8**(3):e58360.

15. Sterling RK, Lissen E, Clumeck N, Sola R, Correa MC, Montaner J, M SS, Torriani FJ, Dieterich DT, Thomas DL *et al*: **Development of a simple noninvasive index to predict significant fibrosis in patients with HIV/HCV coinfection**. *Hepatology (Baltimore, Md)* 2006, **43**(6):1317-1325.

16. Angulo P, Hui JM, Marchesini G, Bugianesi E, George J, Farrell GC, Enders F, Saksena S, Burt AD, Bida JP *et al*: **The NAFLD fibrosis score: a noninvasive system that identifies liver fibrosis in patients with NAFLD**. *Hepatology (Baltimore, Md)* 2007, **45**(4):846-854.

17. Rinella ME, Lazarus JV, Ratziu V, Francque SM, Sanyal AJ, Kanwal F, Romero D, Abdelmalek MF, Anstee QM, Arab JP *et al*: **A multisociety Delphi consensus statement on new fatty liver disease nomenclature**. *Journal of hepatology* 2023, **79**(6):1542-1556.

**Supplement Table**

**Table S1** Weighted baseline characteristics of participants with or without SLF in NHANES

| Variables | | Non-SLF (n = 1969) | SLF (n = 426) | *P* value |
| --- | --- | --- | --- | --- |
| Age (years) | 50.09 (0.74) | 51.75 (1.08) | 0.134 |  |
| Male (%) | 54.78 (1.80) | 63.52 (3.97) | 0.056 |  |
| Race (%) |  |  | 0.286 |  |
| Non-Hispanic Black | 8.41 (1.14) | 8.23 (1.70) |  |  |
| Non-Hispanic White | 62.23 (2.44) | 67.57 (4.55) |  |  |
| Hispanic | 20.34 (2.10) | 16.85 (2.82) |  |  |
| Non-Hispanic Asian | 4.51 (0.73) | 2.85 (0.77) |  |  |
| Other races | 4.52 (0.70) | 4.50 (1.47) |  |  |
| BMI (kg/m^2^) | 33.01 (0.31) | 38.99 (0.49) | <0.001 |  |
| WC (cm) | 109.14 (0.65) | 124.25 (1.12) | <0.001 |  |
| CAP (dB/m) | 319.17 (1.05) | 344.99 (2.57) | <0.001 |  |
| LSM (kPa) | 5.26 (0.05) | 15.43 (1.00) | <0.001 |  |
| PLT (10^6^/L) | 251.77 (2.57) | 239.90 (3.44) | 0.007 |  |
| TBIL (mg/dL) | 0.45 (0.01) | 0.50 (0.03) | 0.054 |  |
| ALT (U/L) | 25.84 (0.56) | 32.80 (1.23) | <0.001 |  |
| AST (U/L) | 21.64 (0.24) | 26.70 (0.94) | <0.001 |  |
| GGT (U/L) | 31.30 (1.00) | 50.01 (2.62) | <0.001 |  |
| ALP (U/L) | 77.71 (0.73) | 81.99 (2.45) | 0.079 |  |
| TP (g/L) | 70.90 (0.18) | 71.26 (0.28) | 0.204 |  |
| ALB (g/L) | 40.94 (0.13) | 40.38 (0.24) | 0.012 |  |
| GLB (g/L) | 29.96 (0.19) | 30.88 (0.29) | 0.005 |  |
| FPG (mg/dL) | 104.38 (0.98) | 117.56 (2.98) | <0.001 |  |
| TG (mg/dL) | 170.37 (4.18) | 193.33 (14.86) | 0.171 |  |
| TC (mg/dL) | 190.36 (1.85) | 183.91 (3.44) | 0.030 |  |
| HDL (mg/dL) | 48.23 (0.45) | 44.22 (0.96) | 0.002 |  |
| LDL (mg/dL) | 108.40 (1.59) | 101.39 (2.79) | 0.009 |  |
| UA (mg/dL) | 5.69 (0.04) | 6.09 (0.14) | 0.004 |  |
| CRE (mg/dL) | 0.89 (0.01) | 0.91 (0.02) | 0.185 |  |
| eGFR (ml/min/m^2^) | 93.45 (0.98) | 91.72 (1.42) | 0.234 |  |
| TyG | 8.91 (0.02) | 9.11 (0.05) | 0.003 |  |
| TyG-BMI | 294.04 (2.62) | 354.29 (4.17) | < 0.001 |  |
| NIKEI | 12.48 (0.17) | 13.14 (0.26) | 0.017 |  |
| FIB-4 | 0.96 (0.03) | 1.15 (0.05) | <0.001 |  |
| NFS | -1.37 (0.06) | -0.39 (0.08) | <0.001 |  |
| Hypertension (%) | 42.41 (2.25) | 50.67 (3.27) | 0.049 |  |
| Diabetes (%) | 21.27 (1.31) | 44.83 (3.38) | <0.001 |  |
| Smoking (%) |  |  | 0.057 |  |
| Never | 57.90 (2.28) | 51.48 (3.44) |  |  |
| Former | 29.07 (2.03) | 37.27 (3.87) |  |  |
| Current | 13.03 (1.50) | 11.25 (2.22) |  |  |
| Alcohol consumption (g/week) | 28.04 (1.40) | 19.64 (2.30) | 0.002 |  |

Note: Continuous variables are shown as mean (SE) and their *P* value was calculated by weighted linear regression model. Categorical values are shown as % (SE) and its *P* value was calculated by weighted chi-square test.

Abbreviations: SE, standard error of mean; MASLD, metabolic dysfunction associated steatotic liver disease; SLF, significant liver fibrosis; NHANES, National Health and Nutrition Examination Survey; CAP, controlled attenuation parameter; LSM, liver stiffness measurements; BMI, body mass index; WC, waist circumference; PLT, platelet; TBIL, total bilirubin; ALT, alanine aminotransferase; AST, aspartate aminotransferase; GGT, γ-glutamyl transpeptidase; ALP, alkaline phosphatase; ALB, albumin; GLB, globulin; TP, total protein; FPG, fasting plasma glucose; TC, total cholesterol; TG, triglyceride; HDL, high-density lipoprotein cholesterol; LDL, low-density lipoprotein cholesterol; UA, uric acid; CRE, creatinine, and eGFR, estimated glomerular filtration rate.

**Table** **S2** Threshold and saturation effect analysis for the relationship between the TyG-BMI and MASLD

| Outcome | Adjusted OR | 95% CI | *P* value |
| --- | --- | --- | --- |
| Model I |  |  |  |
| One line slope | 1.02 | 1.02-1.02 | <0.001 |
| Model II |  |  |  |
| < 180.71 | 1.02 | 1.01-1.02 | <0.001 |
| > 180.71 | 1.13 | 1.07-1.20 | <0.001 |
| *P* for LRT test | <0.001 |  |  |

Note: Model I, linear analysis; Model II, non-linear analysis, LRT, Logarithmic likelihood ratio. The adjustment strategy is the same as the fully adjusted model.

**Table S3** Performance assessment of the TyG, TyG-BMI, HSI, VAI, and ZJU for the prediction of MASLD

| Pairwise comparison | Difference AUC  (95% CI) | *P* value | NRI  (95% CI) | *P* value | IDI  (95% CI) | *P* value |
| --- | --- | --- | --- | --- | --- | --- |
| TyG-BMI vs. TyG | 0.085  (0.071-0.098) | <0.001 | 0.137  (0.107-0.167) | <0.001 | 0.116  (0.091-0.141) | <0.001 |
| TyG-BMI vs. HSI | 0.009  (0.004-0.014) | <0.001 | 0.012  (-0.006-0.030) | 0.196 | 0.015  (-0.006-0.036) | 0.168 |
| TyG-BMI vs. VAI | 0.089  (0.076-0.102) | <0.001 | 0.140  (0.111-0.169) | <0.001 | 0.118  (0.093-0.143) | <0.001 |
| TyG-BMI vs. ZJU | 0.005  (0.002-0.008) | 0.002 | 0.008  (-0.005-0.021) | 0.244 | 0.008  (-0.011-0.027) | 0.399 |

Note: Abbreviations: AUC, area under the receiver operating characteristic curve; NRI, net reclassification index; IDI, integrated discrimination improvement。

**Table** **S4** Threshold and saturation effect analysis for the relationship between the TyG-BMI and SLF

| Outcome | Adjusted OR | 95% CI | *P* value |
| --- | --- | --- | --- |
| Model I |  |  |  |
| One line slope | 1.01 | 1.01-1.01 | <0.001 |
| Model II |  |  |  |
| < 288.59 | 1.01 | 1.01-1.01 | <0.001 |
| > 288.59 | 1.02 | 1.02-1.02 | <0.001 |
| *P* for LRT test | 0.256 |  |  |

Note: Model I, linear analysis; Model II, non-linear analysis, LRT, Logarithmic likelihood ratio. The adjustment strategy is the same as the fully adjusted model.

**Table S5** Performance assessment of the TyG, TyG-BMI, NIKEI, FIB-4, and NFS for the prediction of SLF in MASLD

| Pairwise comparison | Difference AUC  (95% CI) | *P* value | NRI  (95% CI) | *P* value | IDI  (95% CI) | *P* value |
| --- | --- | --- | --- | --- | --- | --- |
| TyG-BMI vs. TyG | 0.158  (0.123-0.193) | <0.001 | 0.239  (0.168-0.310) | <0.001 | 0.071  (0.037-0.105) | <0.001 |
| TyG-BMI vs. NIKEI | 0.152  (0.108-0.197) | <0.001 | 0.237  (0.165-0.309) | <0.001 | 0.069  (0.034-0.104) | 0.024 |
| TyG-BMI vs. FIB-4 | 0.138  (0.093-0.183) | <0.001 | 0.231  (0.151-0.311) | <0.001 | 0.070  (0.034-0.106) | <0.001 |
| TyG-BMI vs. NFS | 0.028  (-0.005-0.061) | 0.098 | 0.064  (-0.001-0.129) | 0.052 | 0.026  (-0.011-0.063) | 0.169 |

Note: Abbreviations: AUC, area under the receiver operating characteristic curve; NRI, net reclassification index; IDI, integrated discrimination improvement。
